# Comparison of the morphine-sparing effect of intraoperative dexmedetomidine with and without loading dose following general anesthesia in multiple-fracture patients: A prospective, randomized, controlled clinical trial

**DOI:** 10.1097/MD.0000000000004576

**Published:** 2016-08-19

**Authors:** Jin-Ning Zhao, Min Kong, Bin Qi, Dong-Jian Ge

**Affiliations:** a Department of Anesthesiology, Sir Run Run Shaw Hospital, School of Medicine, Zhejiang University, Hangzhou; b Department of Anesthesiology, 1882 Middle Ring Road South, Jiaxing, Zhejiang; c Department of Anesthesiology, Huai’an First People's Hospital, Nanjing Medical University, Huai’an, Jiangsu, PR China.

**Keywords:** dexmedetomidine, general anesthesia, morphine, multiple fracture, patient-controlled analgesia

## Abstract

Intraoperative dexmedetomidine (DEX) with or without loading dose both promote morphine-sparing effect in patient-controlled analgesia on postoperative acute pain. However, the contribution of the loading dose to this effect is largely unknown, especially in long-lasting surgeries. The present study was designed to investigate the role of a loading dose of DEX in this morphine-sparing effect in multiple-fracture patients following general anesthesia.

Eighty-six patients scheduled multiple-fracture surgeries under general anesthesia were allocated into 3 groups which were maintained with propofol/remifentanil/Ringer solution (PRR), propofol/remifentanil/DEX with (PRD_w_), or without (PRD_o_) DEX loading dose before induction, respectively. Time to first morphine request and 24-hour morphine consumption was monitored. Pain intensity was evaluated with visual analog scale.

During the first 24 hours following surgery, patients in the PRD_w/o_ group showed increased time to first request of postoperative morphine and decreased total morphine consumption as compared with PRR patients. There was no significant difference with respect to these parameters between patients from the PRD_w_ and PRD_o_ groups. More patients from the PRD_w_ groups experienced intraoperative bradycardia when compared to those from the PRR or PRD_o_ group.

This randomized controlled trial indicates that the morphine-sparing effect of intraoperative DEX was not affected by a loading dose in long-time surgeries.

## Abstract 2

Supplemental Digital Content is available in the text

## 1 Introduction

Acute postoperative pain following multiple fracture is one of the key causes of prolonged convalescence. Opioid, such as morphine, based patient-controlled analgesia (PCA) is well established and has been widely used for postoperative analgesia following different kind of surgeries.^[1,2]^ There has been a continuous pursuit for novel drugs or for more information regarding combining the currently available drugs to reduce the morphine consumption to combat opioid-related side effects.

Dexmedetomidine (DEX), the most selected α2 adrenoceptor agonist with a short terminal half-life (∼2 hours), produces antinociception or proanalgesic effects when combined with other analgesics.^[3]^ It has been reported that perioperative use of DEX lead to lower postoperative pain and reduced opioid consumption in PCA following local and general anesthesia. Therefore, intraoperative DEX might be a novel option for postoperative acute pain control. In general anesthesia, DEX could be used for anesthesia maintenance with or without a loading dose.^[4–9]^ Accumulating recent evidence is showing that intraoperative DEX with or without a loading dose both reduced morphine consumption, also called morphine-sparing effect, in PCA following general anesthesia.^[4–8]^ A loading dose is normally administrated within a very short time period, for example, 5 to 10 minutes, which will result in more hemodynamics alterations, such as decrease of blood pressure and heart rate and intraoperative bradycardia.^[10]^ However, the contribution of the loading dose to the proanalgesic effect of morphine-based PCA was largely unknown.

To address this question, we compare the morphine-sparing effect of intraoperative DEX with and without loading dose multiple fracture under general anesthesia. This study will provide useful information for guiding future use of DEX under general anesthesia, especially in long-lasting surgeries.

## 2 Materials and method

### 2.1 Subjects

**Figure 1:**
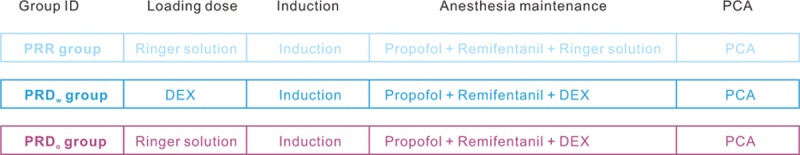
Schematic of anesthesia and postoperative analgesia. Patients received the same treatments for induction and patient-controlled analgesia (Section 2).

**Figure 2:**
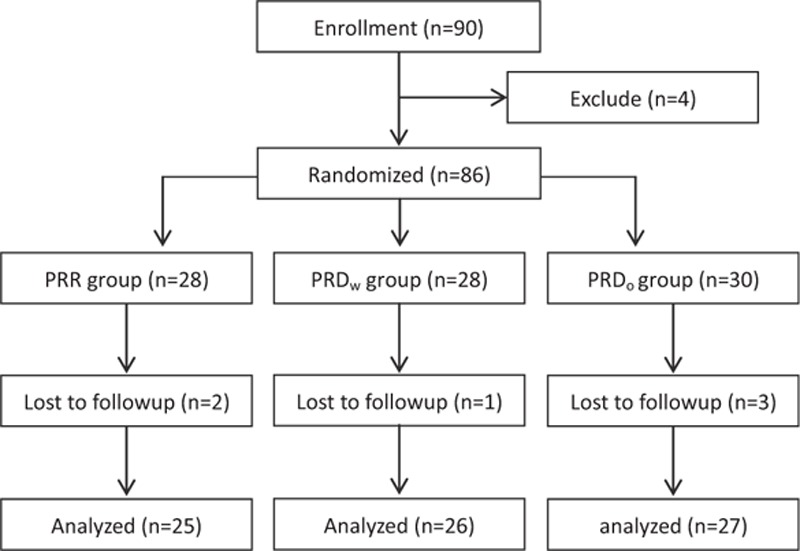
Flow diagram of the study and patient allocation.

This study was registered at chictr.org (ChiCTR-TRC-14004313) and approved by the Institutional Medical Ethics Committee of Nanjing Medical University and was conducted in accordance with the approved guidelines and informed consent from each subject. Eighty-six scheduled for internal fixation surgery under general anesthesia were enrolled and assigned to the PRR (propofol, remifentanil, and Ringer solution for anesthesia maintenance, n = 25, 2 patients were lost because of noncooperation), PRD_w_ group with (propofol, remifentanil, and DEX with a loading dose for anesthesia maintenance, n = 26, 1 patient was lost because of noncooperation), and PRD_o_ group (propofol, remifentanil, and DEX without a loading dose for anesthesia maintenance n = 27, 3 patients were lost because of noncooperation) DEX loading dose using a computer-generated randomized table (Figs. 1 and 2). The maintenance syringe pumps were prepared by a different anesthesiologist to maintain this study as a randomized, double-blinded investigation. Postoperative evaluations were performed by another different anesthesiologist. Patients matching the following criteria were included in this study: between 18 and 65 years old, an American Society of Anesthesiologists (ASA) grade I or II, and weight 45 to 80 kg. Patients were excluded if they had ischemic heart disease; opioid addiction, long-term alcohol abuse, long-term smoking history, sedative-hypnotic drug(s) use; obesity (body mass index [BMI] > 30); a history of postoperative nausea and vomiting; neuropsychiatric diseases or a related treatment history. Patients were instructed in the use of intravenous PCA pump (50 mg morphine and 8 mg ondansetron in 100 mL saline, every pump press resulting in a 2-mL infusion).

### 2.2 Anesthesia

On arrival, electrocardiography, blood pressure, and oxygen saturation were monitored every 5 minutes. Before induction, patients from the PRD_o/w_ group received a fast infusion of 100 mL Ringer solution with or without DEX (1 μg/kg) as a loading dose within 10 minutes. For induction, patients from the 3 groups received midazolam (0.05 mg/kg), remifentanil (2–5 μg/kg), propofol (1.5–2 mg/kg), and cisatracurium (0.2 mg/kg). Immediately after intubation, the patients were ventilated with an oxygen and air mixture (FiO_2_ = 0.4) with a PetCO_2_ of 30 to 35 mm Hg. Intravenous infusion was switched to a maintenance syringe pump at rate of 50 to 80 μg/kg/min for propofol, 0.15 to 0.2 μg/kg/min for remifentanil, and 0.4 μg/kg/h for DEX. Cisatracurium (0.05 mg/kg) was intermittently used for muscle relaxation. The patients were awakened and extubated followed by sedation evaluation using the Ramsay sedation scale (RSS).

### 2.3 Data collection

Demographic information was collected on admission. Hemodynamic parameters were recorded every 5 minutes (data not shown). Ramsay sedation score was evaluated as previous reports.^[6–8]^ Rescue morphine in the postanesthesia care unit (PACU) was included in the total consumption of postoperative PCA morphine. Postoperative acute pain intensity was evaluated with visual analog scale (VAS). PCA pump pressing numbers and adverse effects after surgery were noted.

### 2.4 Statistics

All of the data in the present study were expressed as mean ± standard deviation and analyzed with GraphPad Prism software (San Diego, CA). Parameters such as age, body weight, operation time, anesthesia time, PACU stay time, first-time request, and morphine consumption were analyzed with one-way analysis of variance (ANOVA) followed by Bofferroni posttest. VAS at different time points was analyzed with two-way ANOVA, followed by Bonferroni posttest. ASA grade, female/male ratio, bradycardia incidence, and postoperative adverse effects were analyzed with Fisher test. *P* < 0.05 indicates statistical significance.

## 3 Results

### 3.1 Demographic data and surgery/anesthesia-related information

Patients from the 3 groups had comparable demographic and surgery/anesthesia-related variables, including age, weight, BMI, ASA class, operation time, anesthesia time, and PACU stay time (Table 1).

**Table 1:**
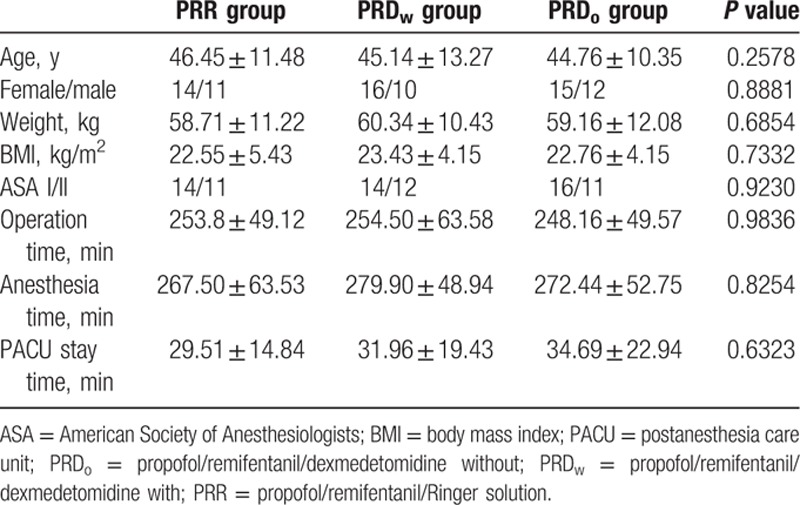
Basic demographic data and surgery/anesthesia-related information. Data shown as mean ± SD.

**Figure 3:**
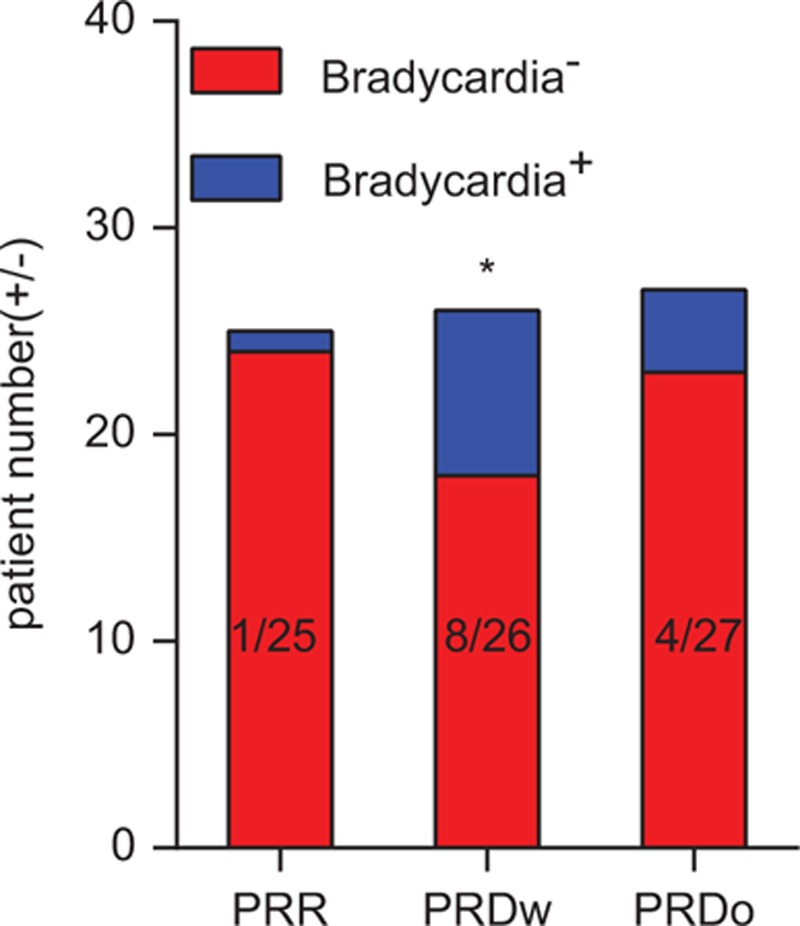
Incidence of bradycardia from 3 different groups. More patients from the propofol/remifentanil/dexmedetomidine with (PRD_w_) group experience intraoperative bradycardia when compared with those from the propofol/remifentanil/Ringer solution (PRR) or propofol/remifentanil/dexmedetomidine without (PRD_o_) group (PRR group vs PRD_W_ group, ∗*P* = 0.0238).

More patients from the PRD_w_ group experience intraoperative bradycardia when compared with those from the PRR or PRD_o_ group (Fig. 3 and Supplementary table 1).

### 3.2 Postoperative sedation evaluation

**Figure 4:**
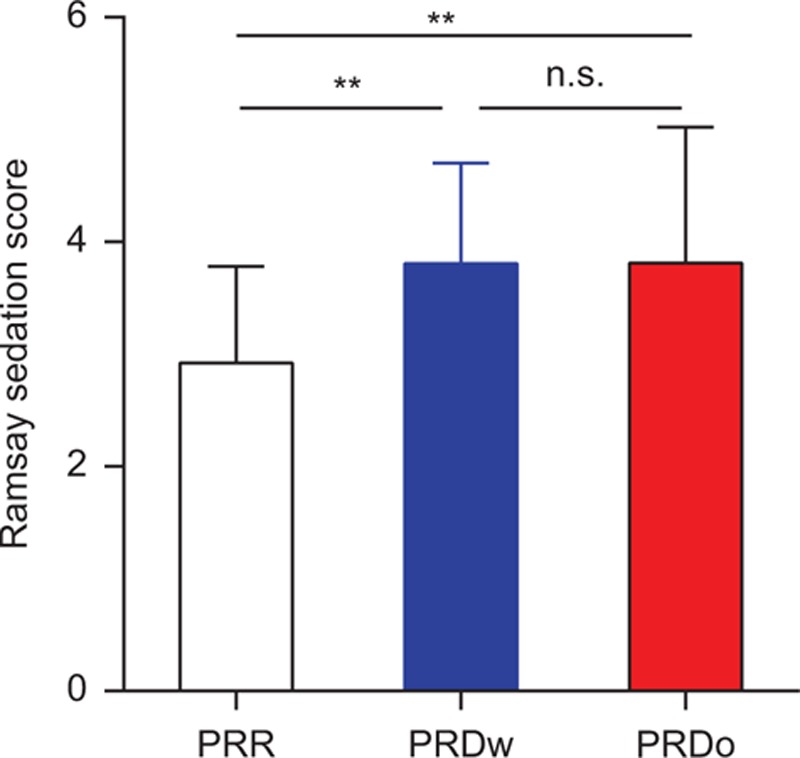
Ramsay sedation score immediately after extubation. Patients from the propofol/remifentanil/Ringer solution (PRR) group displayed a lower Ramsay sedation score when compared with those from the propofol/remifentanil/dexmedetomidine with (PRD_w_) and propofol/remifentanil/dexmedetomidine without (PRD_o_) group (∗∗*P* = 0.0066, PRR group vs PRD_W_ group; ∗∗*P* = 0.0056, PRR group vs PRD_o_ group).

Patients from the PRD_w/o_ groups had a comparable higher immediate Ramsay sedation score after extubation than their controls from the PRR group (Fig. 4 and Supplementary table 2).

### 3.3 Postoperative PCA evaluation

**Figure 5:**
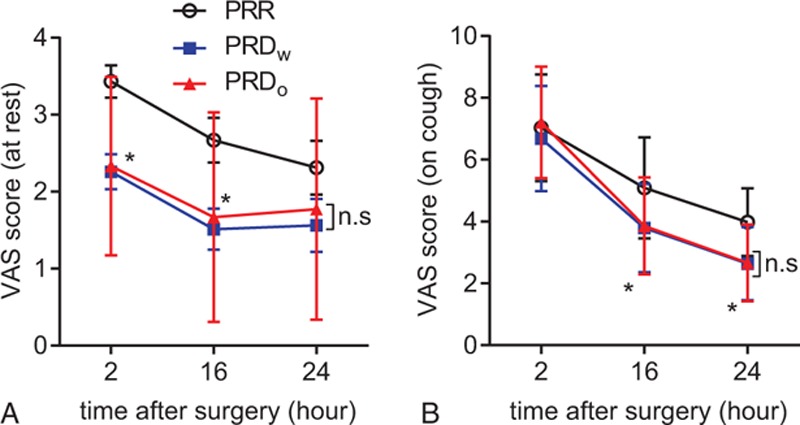
Postoperative visual analog scale (VAS) score at different time points. (A) VAS score at rest (at 2-hour time point: ∗*P* = 0.0299, propofol/remifentanil/Ringer solution [PRR] group vs propofol/remifentanil/dexmedetomidine with [PRD_W_] group; ∗*P* = 0.0476, PRR group vs propofol/remifentanil/dexmedetomidine without [PRD_o_] group. At 16-hour time point: ∗*P* = 0.0323, PRR group vs PRD_W_ group. At 24-hour time point: ∗*P* = 0.5252, PRR group vs PRD_W_ group; ∗*P* > 0.9999, PRR group vs PRD_o_ group). (B) VAS score at movement (at 16-hour time point: ∗*P* = 0.0212, PRR group vs PRD_W_ group; ∗*P* = 0.0305, PRR group vs PRD_o_ group. At 24-hour time point: ∗*P* = 0.0156, PRR group vs PRD_W_ group; ∗*P* = 0.0165, PRR group vs PRD_o_ group).

**Figure 6:**
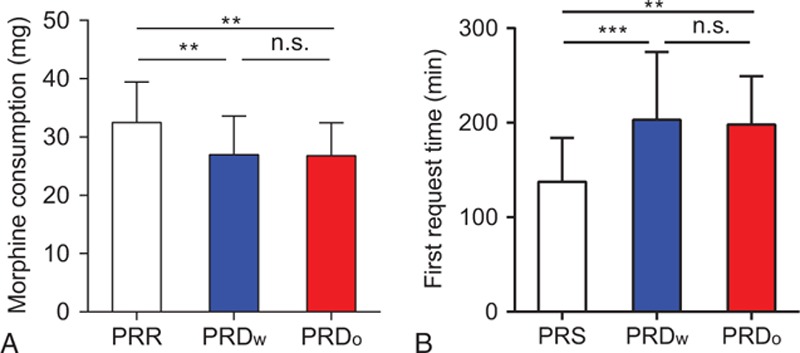
Twenty-four-hour morphine consumption and first request time. (A) Morphine consumption (∗∗*P* = 0.0084, propofol/remifentanil/Ringer solution [PRR] group vs propofol/remifentanil/dexmedetomidine with [PRD_W_] group; ∗∗*P* = 0.0057, PRR group vs propofol/remifentanil/dexmedetomidine without [PRD_o_] group). (B) First morphine request time (∗∗∗*P* = 0.0003, PRR group vs PRD_W_ group. ∗∗*P* = 0.0009, PRR group vs PRD_o_ group).

During the first 24 hours, patients from the PRD groups had a lower VAS score in both the resting (Fig. 5A and Supplementary table 3) and movement state (Fig. 5B and Supplementary table 4) compared to the PRR group. Patients from the 2 PRD groups both had an increased first time of request for postoperative analgesic and reduced the total consumption of morphine during the first postoperative 24 hours (Fig. 6A and B, Supplementary tables 5 and 6). No difference was observed between the patients from PRD_w_ and PRD_o_ groups with respect to these parameters.

### 3.4 Postoperative adverse effects

No differences were observed in postoperative adverse effects among the 3 groups during the first 24 hours (Table 2).

**Table 2:**
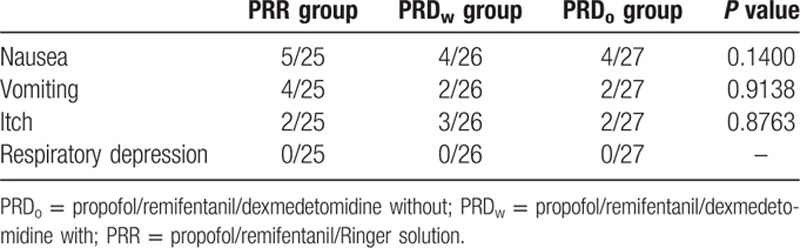
Postoperative side effects from patients in the 2 groups. Data shown as the positive number and percentage of patients.

## 4 Discussion

The present clinical study found that intraoperative DEX with a loading dose showed similar morphine-sparing effect with a higher incidence of bradycardia when compared with intraoperative use of DEX without a loading dose.

It is well known that patients undergoing multiple-fracture surgeries would experience severe acute postoperative pain, which may result in the development of chronic pain state. Opioids, especially morphine, are widely used in PCA pump to alleviate acute pain following these surgeries.^[1,2]^ There has been a continuous pursuit for novel drugs or for more information regarding combining the currently available drugs to reduce the morphine consumption to combat its side effects, such as nausea, vomiting, itching, etc. DEX, a highly α2R agonist developed in the 1990s, was first used as a short-term sedative in the intensive care units.^[3]^ Increasing evidence from clinical studies have reported its potential as an adjuvant for acute pain treatment, mostly in acute perioperative settings. This use suggests that DEX might be used as a novel drug or provide with one more choice to promote the analgesic effect of opioids in surgery-induced acute pain control.^[1]^ For example, recent clinical studies indicated that intraoperative administration of DEX shown potent proanalgesic effect on morphine-based PCA.^[6–8]^ A recent study reported that a combination of DEX and sufentani as PCA displayed a significantly improved analgesic effect in patients following hysterectomy.^[4]^

In the present study, we combined DEX with propofol and remifentanil to maintain the general anesthesia in patients undergoing abdominal surgeries and found that intraoperative use of DEX with or without loading dose both were sufficient to induce a more proanalgesic and morphine-sparing effects. Patients from the PRD group consumed less morphine than those from the PRR group. The analgesic and opioid-sparing effects of DEX have been well described in previous studies both in adults and children.^[6–8,11–13]^ Two recent meta-analyses reported results of reduced morphine consumption and significantly lower pain intensity in patients treated with DEX compared with placebo.^[10,14]^ Consistently, in the present study, there was no difference between PRD_w_ and PRD_o_ groups with respect to the proanalgesic and morphine-sparing effect. Together with these previous findings, we further confirmed that intraoperative use of DEX with or without loading dose both were sufficient to promote morphine-based PCA following abdominal surgery.

There are several possible mechanisms underlying the long-term analgesic effect: unlike with the sedation effect, DEX uses a different α2AR-dependent downstream mechanism to act as an analgesic. Another reason might be that DEX prolongs the analgesic time and analgesic effect of other analgesics.^[3]^ Although an animal study reported that its analgesic properties could be neutralized by the α2AR antagonist,^[15]^ we cannot completely exclude the remote possibility that DEX also uses α2AR-independent mechanisms to exert its analgesic effects. In the present study, the long-lasting operation time (>4 hours) might dilute the postoperative effect of the loading dose because of a very short half-time of DEX (∼2 hours).^[1]^

The most interesting part of the study is that there is no difference between the patients from the 2 PRD groups which received Ringer solution or DEX as a loading dose before induction. The multiple-fracture surgeries in this study lasted for longer than 4 hours which may be longer enough to allow intraoperative DEX to reach the effective concentration to promote the analgesic effect of morphine. DEX induces hemodynamic changes, such as hypertension, hypotension, and bradycardia, especially after a loading dose. As reported by the 2 meta-analyses discussed above,^[10,14]^ we saw more patients experienced bradycardia following the administration of a DEX loading dose. These data supported that intraoperative use of DEX without loading dose might be an useful and better choice to induce a morphine-sparing effect on PCA following multiple-fracture surgeries lasting longer than 4 hours. Similar comparison between the intraoperative use of DEX with and without loading dose should also be performed in short-time surgeries.

RSS, Richmond Agitation Sedation Scale (RASS), Sedation Agitation Scale (SAS), and Adaption to Intensive Care Environment Scale are 4 commonly used and sufficient for sedation evaluation. A recent study performed in intensive care unit mechanically ventilated patients indicated that RSS and RASS could be used for monitoring the depth of sedation based on their higher correlation with bispectral index values.^[16]^ Both RSS and RASS are widely used for sedation monitoring postoperatively. We believe that there might be geographical preference of the use of these 2 methods. For example, we found some anesthesiologists from India used RSS,^[17,18]^ RASS and SAS are more preferred in North America, and some anesthesiologists used both from Turkey.^[16]^ And also, we do believe that there could be personal preference among anesthesiologists.

There might be limitations in the present study: we used a VAS for postoperative pain evaluation. The Numerical Rating Scale (NRS) is another well established and widely used method for pain evaluation, and it was reported to be more reliable than the VAS in some cases.^[19]^ Our hospital is located on the demarcation line between North and South China, and we received patients from different provinces. The heavy accents with which some patients spoke might have been a limitation to the use of the NRS. For example, some patients from South China often pronounce the number “10” (“Shi” in Chinese mandarin) as “Si” (which is the pronunciation of the number “4”). Furthermore, the anesthesiologists who performed this study also came from different provinces of the country. Thus, to avoid misunderstanding, we used the VAS to evaluate postoperative pain. We nevertheless encourage the NRS to be used in future studies if the conditions are applicable, because it is easier to perform, saves more time, and is more reliable than the VAS. As we described above, we only performed this comparison in surgeries with anesthesia time longer than 4 hours, similar comparison should be repeated in short-term surgeries.

Taken together, maintenance with DEX without loading dose shown similar proanalgesic and morphine-sparing effects without hemodynamic alterations, such as bradycardia, induced by DEX loading dose.

## Supplementary Material

Supplemental Digital Content
