# Supplementary material for: Comparison of the morphine-sparing effect of intraoperative dexmedetomidine with and without loading dose following general anesthesia in multiple-fracture patients: A prospective, randomized, controlled clinical trial
Source: Medicine (Baltimore). 2016 Aug 19;95(33):e4576. doi: 10.1097/MD.0000000000004576 (PMC5370813; doi:10.1097/MD.0000000000004576)
Supplement: Supplemental Digital Content [file medi-95-e4576-s001.docx]

**supplementary table 1. raw data of Figure 3.**

|  | PRR group | PRD_w_ group | PRD_o_ group |
| --- | --- | --- | --- |
| Bradycardia (+/-) | 1/24 | 8/18^*^ | 4/23 |

*P=0.0238, PRR group v.s. PRD_W_ group.

P=0.3517, PRR group v.s. PRD_o_ group.

P=0.2021, PRD_w_ group v.s. PRD_o_ group.

**supplementary table 2: raw data of Figure 4.**

|  | PRR group | PRD_w_ group | PRD_o_ group |
| --- | --- | --- | --- |
| Ramsay score | 2.92 ± 0.8622 | 3.808 ± 0.8953** | 3.815 ± 1.21** |

**P=0.0066, PRR group v.s. PRD_W_ group.

**P=0.0056, PRR group v.s. PRD_o_ group.

P=0.9996, PRD_w_ group v.s. PRD_o_ group.

**supplementary table 3: raw data of Figure 5A.**

|  | PRR group | PRD_w_ group | PRD_o_ group |
| --- | --- | --- | --- |
| 2h | 3.43 ± 1.05 | 2.26 ± 1.16* | 2.33 ± 1.16* |
| 16h | 2.67 ± 1.45 | 1.51 ± 1.36* | 1.67 ± 1.36 |
| 24h | 2.31 ± 1.76 | 1.56 ± 1.76 | 1.77 ± 1.44 |

at 2h time point:

*P=0.0299, PRR group v.s. PRD_W_ group.

*P=0.0476, PRR group v.s. PRD_o_ group.

P>0.9999, PRD_w_ group v.s. PRD_o_ group.

at 16h timepoint:

*P=0.0323, PRR group v.s. PRD_W_ group.

P=0.1006, PRR group v.s. PRD_o_ group.

P>0.9999, PRD_w_ group v.s. PRD_o_ group.

at 24 hour timepoint:

*P=0.5252, PRR group v.s. PRD_W_ group.

*P>0.9999, PRR group v.s. PRD_o_ group.

P>0.9999, PRD_w_ group v.s. PRD_o_ group.

**supplementary table 4: raw data of Figure 5B.**

|  | PRR group | PRD_w_ group | PRD_o_ group |
| --- | --- | --- | --- |
| 2h | 7.03 ± 1.73 | 6.68 ± 1.70 | 7.20 ± 1.80 |
| 16h | 5.09 ± 1.64 | 3.79 ± 1.43* | 3.85 ± 1.57* |
| 24h | 3.98 ± 1.09 | 2.54 ± 1.19* | 2.66 ± 1.24* |

at 2h time point:

P>0.9999, PRR group v.s. PRD_W_ group.

P>0.9999, PRR group v.s. PRD_o_ group.

P>0.9999, PRD_w_ group v.s. PRD_o_ group.

at 16h timepoint:

*P=0.0212, PRR group v.s. PRD_W_ group.

*P=0.0305, PRR group v.s. PRD_o_ group.

P>0.9999, PRD_w_ group v.s. PRD_o_ group.

at 24 hour timepoint:

*P=0.0156, PRR group v.s. PRD_W_ group.

*P=0.0165, PRR group v.s. PRD_o_ group.

P>0.9999, PRD_w_ group v.s. PRD_o_ group.

supp**lementary table 5: raw data of Figure 6A.**

|  | PRR group | PRD_w_ group | PRD_o_ group |
| --- | --- | --- | --- |
| Morphine consumption (mg) | 32.48 ± 0.6959 | 26.86 ± 0.663** | 26.78 ± 5.673** |

**P=0.0084, PRR group v.s. PRD_W_ group.

**P=0.0057, PRR group v.s. PRD_o_ group.

P=0.9940, PRD_w_ group v.s. PRD_o_ group.

**supplementary table 6: raw data of Figure 6B.**

|  | PRR group | PRD_w_ group | PRD_o_ group |
| --- | --- | --- | --- |
| First request time (min) | 137.6 ± 46.33 | 203.1 ± 71.72*** | 198.1 ± 51.03** |

***P=0.0003, PRR group v.s. PRD_W_ group.

**P=0.0009, PRR group v.s. PRD_o_ group.

P=0.9470, PRD_w_ group v.s. PRD_o_ group.
